# Evaluation of the in vitro acaricidal activity of ethanol extracts of seven Chinese medicinal herbs on *Ornithonyssus sylviarum* (Acari: Macronyssidae)

**DOI:** 10.1007/s10493-022-00716-9

**Published:** 2022-06-23

**Authors:** Yichen Jian, Shijie Li, Dongliang Li, Changshen Ning, Sumei Zhang, Fuchun Jian, Hongbin Si

**Affiliations:** 1 College of Animal Veterinary Medicine, Henan Agricultural University, Zhengzhou, 450046 China; 2 College of Animal Science and Technology, GuangXi University, Nanning, 530000 China

**Keywords:** Chinese herbal medicine, *Ornithonyssus sylviarum*, Ethanol extract, Acaricidal activity, Northern fowl mite

## Abstract

*Ornithonyssus sylviarum* (Acari: Macronyssidae) is a common ectoparasite that feeds on the blood of poultry. Following infestation, this mite will cause symptoms such as weight loss, anemia, and decreased egg production. To explore green and safe drugs for the prevention and treatment of *O. sylviarum*, this study evaluated the effects of ethanol extracts of seven Chinese medicinal herbs—*Leonurus artemisia* (motherwort), *Illicium verum* (star anise), *Cinnamomum cassia* (cinnamon), *Hibiscus syriacus*, *Artemisia argyi* (Chinese mugwort), *Taraxacum* sp. (dandelion), and *Syzygium aromaticum* (clove)—on *O. sylviarum* at different life stages. The results showed that different methods of administration affected the acaricidal efficacy of these plant extracts on *O. sylviarum*. After 6 h of administration with the fumigation method, the acaricidal efficacy of *S. aromaticum* on adults, nymphs and larvae of *O. sylviarum* reached 100%. 30 min after administration with the infiltration method, *S. aromaticum*, *H. syriacus* and *L. artemisia* showed acaricidal effects on adults and nymphs of *O. sylviarum* reaching 100%. In another experiment evaluating the inhibition of egg hatching of *O. sylviarum* with alcohol extracts of these seven herbs, at 48 h after treatment, *A. argyi* and *C. cassia* showed inhibition rates of 19.4%. The results of this study indicate that *S. aromaticum* induced mortality at all stages of *O. sylviarum*, whereas *A. argyi* was found to be the most effective at inhibiting the mite’s egg hatching among the seven herbs. These herbs can therefore be used as potential substitutes for chemical pesticides to prevent and control *O. sylviarum*. These results provide practical knowledge for the control of *O. sylviarum*.

## Introduction

The northern fowl mite (NFM), *Ornithonyssus sylviarum* (Canestrini & Fanzago) (Acari: Macronyssidae), is a permanent ectoparasite that feeds on the blood of birds (incl. poultry) and humans. The NFM often parasitizes the vent region of the host and completes its entire life cycle on the host (Murillo and Mullens 2017). Although there have been no reports of NFM bites on humans, *Dermanyssus gallinae* and *Ornithonyssus bursa* bites have been reported, and people who are bitten can experience skin diseases such as skin itching, rash, dermatitis and urticaria (Castelli et al. 2015; Sioutas et al. 2021). Infested birds have clinical manifestations of restlessness, feather pecking, skin inflammation, anemia, slowed growth, and decreased egg production and quality (Murillo et al. 2020; Vezzoli et al. 2016). In severe infestations, the number of NFMs on a chicken can reach 50,000, a level that will cause the infested chickens to lose up to 6% of their blood every night (DeVaney 1981). The NFM is also one of the most prevalent ectoparasites on poultry farms in most parts of the world (Jansson et al. 2014; Knee and Proctor 2007; Waap et al. 2017). According to previous reports, > 70 species of wild birds in North America can serve as hosts of the NFM, including starlings and house sparrows (Knee and Proctor 2007). China is considered the world's largest egg producer, and poultry farming is an important industry in this country. According to an epidemiological survey of ectoparasites in China (Wang et al. 2010), 91.3% of poultry farms are infested with ectoparasites, among which *O. sylviarum* accounted for 46.9%.

At present, the most common method used to control ectoparasites is the spraying of chemical insecticides. In China, pyrethroids, organophosphates, cypermethrin or other acaricides are used to control ectoparasites in 95% of laying hens and 74.9% of breeders (Wang et al. 2010). There are two problems with spraying acaricides to prevent and control NFMs. First, NFMs can survive for several weeks in an environment without a host, and chemical acaricides must be applied to large areas in chicken coops, which is likely to create problems with chemical residues (Gokbulut et al. 2019). In addition, mites are small, and they attach to the roots of chicken feathers to lay eggs and reproduce, causing the feathers to become densely covered with mite bodies and a large quantity of mite feces. When infestations are high, the feathers soiled by the mites will stick together, which greatly increases the difficulty of applying medication. In this situation, it is necessary to apply the acaricides multiple times, increasing the probability of pesticide resistance, which generally leads to problems such as decreased efficacy (Marangi et al. 2009). To reduce the use of chemical pesticides and address the problems associated with insecticide residues and insecticide resistance, physical and biological methods have been used in some European countries for prevention and control, including the use of inert dust, diatomaceous earth, synthetic silica (Kilpinen and Steenberg 2009), and fungi such as *Metarhizium anisopliae*, *Beauveria bassiana*, *Aspergillus oryzae* and others (Wang et al. 2019; Steenberg and Kilpinen 2014). Furthermore, natural plant essential oils have shown promise as substitutes for synthetic pesticides. According to reports, many plant essential oils, such as carvacrol, thymol, and the essential oils of fennel and lemongrass, have shown in vitro efficacy against *D. gallinae* and *Sarcoptes scabiei* (Baran et al. 2020; Li et al. 2020; Nechita et al. 2015).

To explore safe and effective potential insecticides, extracts of seven plants used as Chinese herbal medicines, including those obtained from clove and motherwort, were used in mortality tests against NFMs.

## Materials and methods

### Herbal preparations

The test plants clove, cinnamon, hibiscus, Chinese mugwort, dandelion, star anise, and motherwort were all purchased from Tongrentang Chinese Herbal Medicine Wholesale Store (Beijing, China; Table 1). The positive controls (5% cypermethrin and 100 mg/mL ivermectin) were purchased from Henan Anjin Biotechnology (Xinxiang, Henan, China). Anhydrous ethanol (analytical grade) was purchased from Fuyu Fine Chemical (Tianjin, China). The negative control (0.9% sodium chloride) was purchased from Henan Kelun Pharmaceutical (Anyang, Henan, China).

**Table 1:** Information on the seven Chinese medicinal herbs used in the study

Name	Family	Species	Tested part
Motherwort	Labiatae	*Leonurus artemisia* L.	Whole plant
Star anise	Magnoliaceae	*Illicium verum* Hook. f.	Ripe fruit
Cinnamon	Lauraceae	*Cinnamomum cassia* Presl	Bark
Hibiscus	Malvaceae	*Hibiscus syriacus* L.	Flowers
Chinese mugwort	Asteraceae	*Artemisia argyi* H.Lév. & Vaniot	Whole plant
Dandelion	Asteraceae	*Taraxacum* sp.	Whole plant
Clove	Myrtaceae	*Syzygium aromaticum* (L.)	Whole plant

### Ethanol extraction of herbal medicines

Fifty grams of the above herbs was crushed and passed through 20-mesh screens. Next, 200 mL of a 90% ethanol solution was added; the samples were soaked for 1 week and then filtered through six layers of gauze. The residue was then added to 100 mL of a 90% ethanol solution and soaked again for 24 h. This solution was filtered through six layers of gauze, and then the two filtrates were combined. The filtrate was centrifuged at 3000 rpm for 10 min, and the ethanol was evaporated from the supernatant in a boiling water bath to concentrate it into a paste. This paste was diluted to 50 mL with 0.9% sodium chloride; i.e., 50 g of each herb was used to make 50 mL of the experimental insecticide. This insecticide was then stored at 4 °C for later use.

### Acquisition of mites

**Fig. 1:**
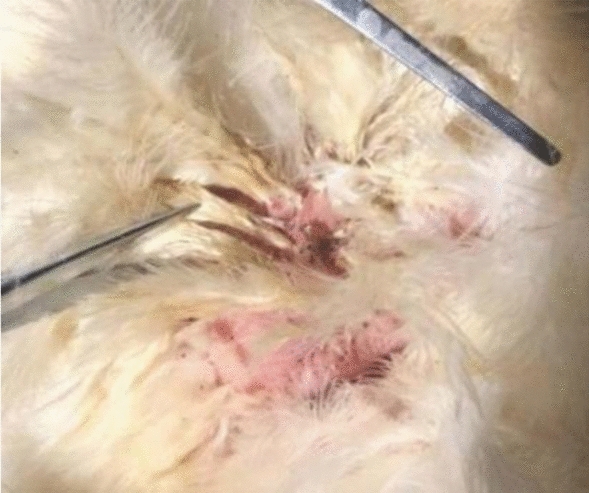
Parasitic mites (northern fowl mite, *Ornithonyssus sylviarum*) near the anus of a chicken

A suspected case of chicken mite infestation occurred at a chicken farm in Yuanyang County, Xinxiang city, Henan Province, China, with dense, fast-moving mites appearing in the vent region of the sick chickens (Fig. 1). A small brush was used to transfer the mites into a No. 4 seal bag, which was taken to the Parasitology Laboratory of Henan Agricultural University. Under a stereomicroscope, using the identification method of Di Palma et al. (2012), the morphology of the mouth organs, horns, and vent region of the mites was examined, and the molecular method of Bhowmick et al. (2019) was used for further identification. The final identification confirmed the presence of *O. sylviarum*. *Ornithonyssus sylviarum* was then transferred to a 9-cm Petri dish, and a layer of Vaseline was applied around the dish to prevent the mites from crawling out until they were used in subsequent trials.

### Infiltration method

All herbal medicine extracts were diluted to 0.5 g/mL. Ten viable mites were selected under a stereomicroscope (mites were grouped into adults + nymphs and larvae, 10 mites in both groups) and placed into the wells of a 24-well cell culture plate (Nest Biotechnology, Shanghai, China), to which 0.3 mL of an herbal extract (0.5 g/mL) was added. There were three replicates per set of experiments. The negative control was treated with 0.9% NaCl, and the positive controls were treated with 100 mg/mL ivermectin and 5% cypermethrin. The wells were observed for 3 h, and the numbers of dead and surviving mites were recorded at 0.5 h, 1 h and 3 h. If a mite remained immobile after being continuously stimulated with a needle for 1 min, it was considered to be dead.

### Fumigation

All herbal medicine extracts were diluted to 0.5 g/mL, and healthy viable mites at different stages (adults + nymphs are in a group, and larvae in another, 10 mites in both groups) were selected under a stereomicroscope and placed in a 2-mL Eppendorf tube. Each tube was sealed with a cotton ball to which 0.8 mL of herbal liquid (0.5 g/mL) had been added. At the beginning of the evaluation of each herb extract, the negative control was treated with 0.9% NaCl, and the positive controls were treated with 100 mg/mL ivermectin and 5% cypermethrin. Three replicates were used for each set of experiments. Mite mortality was observed at 6 h, 12 h, and 24 h under a stereomicroscopy. If a mite remained immobile after being continuously stimulated with a needle for 1 min, it was considered to be dead.

### In vitro evaluation of the ovicidal activity of alcohol extracts of herbs

Twenty healthy, viable female adults were selected and transferred to 36-well cell culture plates (Nest Biotechnology). The plates were placed in a constant temperature and humidity incubator, and observed every 24 h to collect fresh, healthy eggs. The incubator was at 26 °C, 65–70% RH, and completely dark. Groups of 10 eggs were collected. There were three replicates of each group. A 0.2 mL aliquot of the diluted herbal liquid (0.5 g/mL) was placed in full contact with the eggs for 3 min; the eggs were then transferred to fresh non-herbal solution in a 36-well cell culture plate, and the plate was placed in a constant-temperature and -humidity incubator. Observations were made at 24 h and 48 h. Positive and negative controls were also established. The positive controls were 100 mg/mL ivermectin and 5% cypermethrin, whereas the negative control was 0.9% NaCl. A lower hatching rate indicates a stronger inhibition of egg hatching.

### Statistical analysis

The experimental data were sorted with Excel, and mortality of the test group was calculated: mortality (%) = [no. dead mites/total no. tested mites] × 100%. The mortality in the treatment groups was corrected to take into account control mortality using Abbott’s formula (Abbott 1925): corrected mortality (%) = [1 – % dead mites in the treated plate/% dead mites in the untreated control plate] × 100%. SPSS v.20.0 was used to perform one-way ANOVA on the experimental data, followed by least significant difference (LSD) tests. Prism v.8.0 software was used for drawing figures (GraphPad, San Diego, CA, USA).

## Results

### Effects of herbal extracts on mite mortality through infiltration

#### Adult mites/nymphs

After 30 min of treatment, the mortality of adult and nymphal mites associated with clove, hibiscus and motherwort reached 100%, similar to the mortality in the positive control groups (ivermectin, 100%; cypermethrin, 93%); these values were significantly higher than those associated with Chinese mugwort (77%), dandelion (73%), star anise (57%), and cinnamon (13%) (Table 2). No mortality occurred in the negative control group. After 1 h of treatment, the mortality rate associated with cypermethrin in the positive control reached 100%, which was significantly higher than that observed for the extracts of cinnamon (83%), Chinese mugwort (80%), dandelion and star anise (both 73%) (Table 2). After 3 h, the corrected mortality rate associated with Chinese mugwort reached 100%, and the mortality rates associated with dandelion and cinnamon (both 85%) were significantly higher than that observed for star anise (69%) and the negative control group (13%) (Table 2).

**Table 2:** Mean (± SD) corrected mortality (%) of northern fowl mite (NFM) adults + nymphs and larvae exposed for 0.5 h, 1 h and 3 h to alcohol extracts of seven Chinese medicinal herbs via infiltration (cypermethrin and ivermectin act as positive controls, 0.9% NaCl as a negative control)

NFM stage	Treatment	n	30 min	1 h	3 h
Adults/nymphs	Clove	30	100a	100a	100a
	Hibiscus	30	100a	100a	100a
	Motherwort	30	100a	100a	100a
	Chinese mugwort	30	76.6 ± 5.8b	80.0 ± 17.3b	100a
	Dandelion	30	73.3 ± 5.8b	73.3 ± 5.8b	84.5 ± 5.8b
	Star anise	30	56.6 ± 11.5c	73.3 ± 5.8b	69.2 ± 5.8b
	Cinnamon	30	13.3 ± 5.8d	83.3 ± 5.8b	84.5 ± 11.5b
	5% cypermethrin	30	93.3 ± 5.8a	100a	100a
	100 mg/mL ivermectin	30	100a	100a	100a
	Negative control	30	0d	0c	13.3 ± 5.8c
Larvae	Clove	30	100a	100a	100a
	Hibiscus	30	90.0 ± 10.0a	93.3 ± 5.8a	100a
	Motherwort	30	100a	100a	100a
	Chinese mugwort	30	46.6 ± 15.3c	63.3 ± 5.8b	70.0 ± 10.0b
	Dandelion	30	90.0 ± 17.3a	90.0 ± 17.3a	90.0 ± 17.3a
	Star anise	30	0d	3.3 ± 5.8c	6.6 ± 5.8c
	Cinnamon	30	50.0 ± 10.0c	60.0 ± 10.0b	63.3 ± 5.8b
	5% cypermethrin	30	70.0 ± 17.3b	70.0 ± 17.3b	70.0 ± 17.3b
	100 mg/mL ivermectin	30	100a	100a	100a
	Negative control	30	0d	0c	0c

n—total number of mites tested

Means within a column and within a mite stage followed by different letters are significantly different (one-way ANOVA followed by LSD tests: P < 0.01)

#### Larvae

After 30 min of treatment, the larval mortality caused by clove, motherwort and ivermectin (positive control) reached 100%, whereas that caused by hibiscus and dandelion reached 90%—all were significantly higher than the mortality observed for cypermethrin (70%), cinnamon (50%), and Chinese mugwort (47%) (Table 2); star anise and the negative control caused no larval mortality at all. After 1 h of treatment, the mortality associated with hibiscus (93%) and dandelion (90%) was significantly higher than that associated with cypermethrin (70%), Chinese mugwort (63%), and cinnamon (60%); there was no statistical significance between the star anise group (3%) and the negative control group (0%) (Table 2). After 3 h, the mortality caused by hibiscus reached 100% and that caused by dandelion 90%, both significantly higher than the mortality caused by cypermethrin, Chinese mugwort (both 70%), cinnamon (63%) and star anise (7%) (Table 2). The mortality associated with cypermethrin (70%) was significantly higher than that associated with star anise (7%) and the negative control (0%). There was no significant difference between star anise (7%) and the negative control (0%) (Table 2).

### Effects of herbal extracts on mite mortality through fumigation

#### Adult mites/nymphs

After 6 h of treatment, the corrected mortality rates of adult and nymphal mites associated with clove and ivermectin (positive control) reached 100%. In addition, the mortality rates associated with hibiscus (93%) and cinnamon (86%) were significantly higher than those associated with Chinese mugwort (76%), motherwort (61%), star anise (10%), dandelion (7%) and cypermethrin (second positive control, 0%) (Table 3). Among these extracts, the corrected mortality rates associated with star anise, dandelion and cypermethrin did not significantly differ from that of the untreated control group (3%). After 12 h of treatment, the corrected mortality rates associated with hibiscus (97%), cinnamon (97%), and Chinese mugwort (90%) were significantly higher than those associated with motherwort (74%), star anise (21%), dandelion (10%), cypermethrin (0%) and the negative control (3%) (Table 3). After 24 h, Chinese mugwort (89%) and motherwort (73%) caused significantly higher corrected mortality than star anise (25%), dandelion (14%), cypermethrin (–4%) and the negative control (7%), whereas the other groups reached 100% mortality (Table 3).

**Table 3:** Mean (± SD) corrected mortality (%) of northern fowl mite (NFM) adults + nymphs and larvae at 6 h, 12 h and 24 h after application of alcohol extracts of seven Chinese medicinal herbs via fumigation (cypermethrin and ivermectin act as positive controls, 0.9% NaCl as a negative control)

NFM stage	Treatment	n	6 h	12 h	24 h
Adults/nymphs	Clove	30	100a	100a	100a
	Hibiscus	30	93.1 ± 5.8a	96.5 ± 5.8a	100a
	Motherwort	34	60.8 ± 9.6bc	73.5 ± 11.2b	72.6 ± 11.2b
	Chinese mugwort	30	75.8 ± 5.8bc	89.7 ± 10.0a	89.3 ± 10.0a
	Dandelion	30	6.9 ± 0.0d	10.3 ± 5.8 cd	14.3 ± 0.0c
	Star anise	30	10.3 ± 5.8d	20.7 ± 5.8c	25.1 ± 0.0c
	Cinnamon	30	86.1 ± 15.3ab	96.5 ± 5.8a	100a
	5% cypermethrin	30	0.0 ± 5.8d	0.0 ± 5.8d	–3.5 ± 5.8d
	100 mg/mL ivermectin	30	100a	100a	100a
	Negative control	30	3.3 ± 5.8d	3.3 ± 5.8d	6.6 ± 5.8d
Larvae	Clove	30	100a	100a	100a
	Hibiscus	30	60.0 ± 10.0b	86.1 ± 11.5ab	100a
	Motherwort	30	53.3 ± 5.8b	60.0 ± 15.3b	86.1 ± 5.8ab
	Chinese mugwort	30	6.6 ± 5.8c	13.8 ± 15.3c	24.1 ± 15.3c
	Dandelion	30	60.0 ± 10.0b	72.4 ± 5.8b	93.1 ± 11.5a
	Star anise	30	0c	8.0 ± 17.3c	27.6 ± 17.3c
	Cinnamon	30	93.3 ± 11.5a	96.5 ± 5.8a	100a
	5% cypermethrin	30	3.3 ± 5.8c	8.0 ± 10.0c	11.5 cd
	100 mg/mL ivermectin	30	100a	100a	100a
	Negative control	30	0c	3.3 ± 5.8c	3.3 ± 5.8d

n—total number of mites tested

Means within a column and within a mite stage followed by different letters are significantly different (one-way ANOVA followed by LSD tests: P < 0.01)

#### Larvae

After 6 h of treatment, the larval mortality associated with clove and ivermectin reached 100%, whereas the mortality associated with cinnamon (93%) was significantly higher than that associated with hibiscus, dandelion (both 60%), and motherwort (53%) (Table 3). The mortality caused by Chinese mugwort (7%), cypermethrin (3%) and star anise (0%) did not differ from that caused by the negative control (0%). After 12 h of treatment, cinnamon (97%) and hibiscus (86%) caused significantly higher corrected mortality than dandelion (72%), motherwort (60%), and Chinese mugwort (14%), whereas there were no significant differences in corrected mortality associated with cypermethrin, star anise (both 8%) and the negative control (3%) (Table 3). After 24 h, the corrected mortality associated with dandelion (93%) and motherwort (86%) was significantly higher than that associated with Chinese mugwort (24%) and star anise (28%), whereas cypermethrin (12%) and the negative control (3%) showed no significant differences. The other herb extracts caused 100% mortality (Table 3).

### In vitro evaluation of the ovicidal activity of herb extracts

After 24 h, the lowest hatching rates were observed of motherwort, Chinese mugwort and dandelion (9%, 10% and 13%, respectively) followed by cinnamon and clove (16% and 20%, respectively); the results for these herbs were significantly different from those for the negative control (43%) (Table 4). There were no significant differences in the hatchability rates of eggs exposed to star anise and hibiscus (both 37%), or the negative control (43%). The hatchability of eggs exposed to cypermethrin and ivermectin (both 13%) were significantly lower than that of star anise and hibiscus (both 37%) and negative control (43%) (Table 4). After 48 h of treatment, significant differences were observed between all treatment groups and the negative control group (73%) (Table 4). The lowest hatching rates of mite eggs were observed in association with Chinese mugwort and cinnamon (both 19%), whereas clove, cypermethrin (both 23%), and motherwort (31%) had significantly stronger inhibitory effects than dandelion (37%), ivermectin (38%), star anise (40%), and hibiscus (53%) (Table 4).

**Table 4:** Mean (± SD) hatching rate (%) of northern fowl mite (NFM) eggs after 24 h and 48 h of application of alcohol extracts of seven Chinese medicinal herbs via fumigation (cypermethrin and ivermectin act as positive controls, 0.9% NaCl as a negative control)

Treatment	n	24 h	48 h
Chinese mugwort	32	9.66 ± 10.01a	19.39 ± 10.05a
Cinnamon	31	16.11 ± 6.73a	19.44 ± 10.84a
Cloves	31	19.69 ± 10.45a	22.72 ± 6.36a
Motherwort	32	9.23 ± 10.08a	31.02 ± 8.51a
Dandelion	30	13.33 ± 5.77a	36.66 ± 5.77b
Star anise	30	36.66 ± 15.27b	40.00 ± 20.00b
Hibiscus	30	36.66 ± 15.27b	53.33 ± 5.77b
100 mg/mL ivermectin	31	13.33 ± 11.54ac	38.48 ± 7.83bc
5% cypermethrin	30	13.33 ± 15.27ac	23.33 ± 15.27ad
Negative control	30	43.33 ± 5.77bd	73.33 ± 11.54e

n—total number of eggs tested

Means within a column followed by different letters are significantly different (one-way ANOVA followed by LSD tests: P < 0.01

## Discussion

**Fig. 2:**
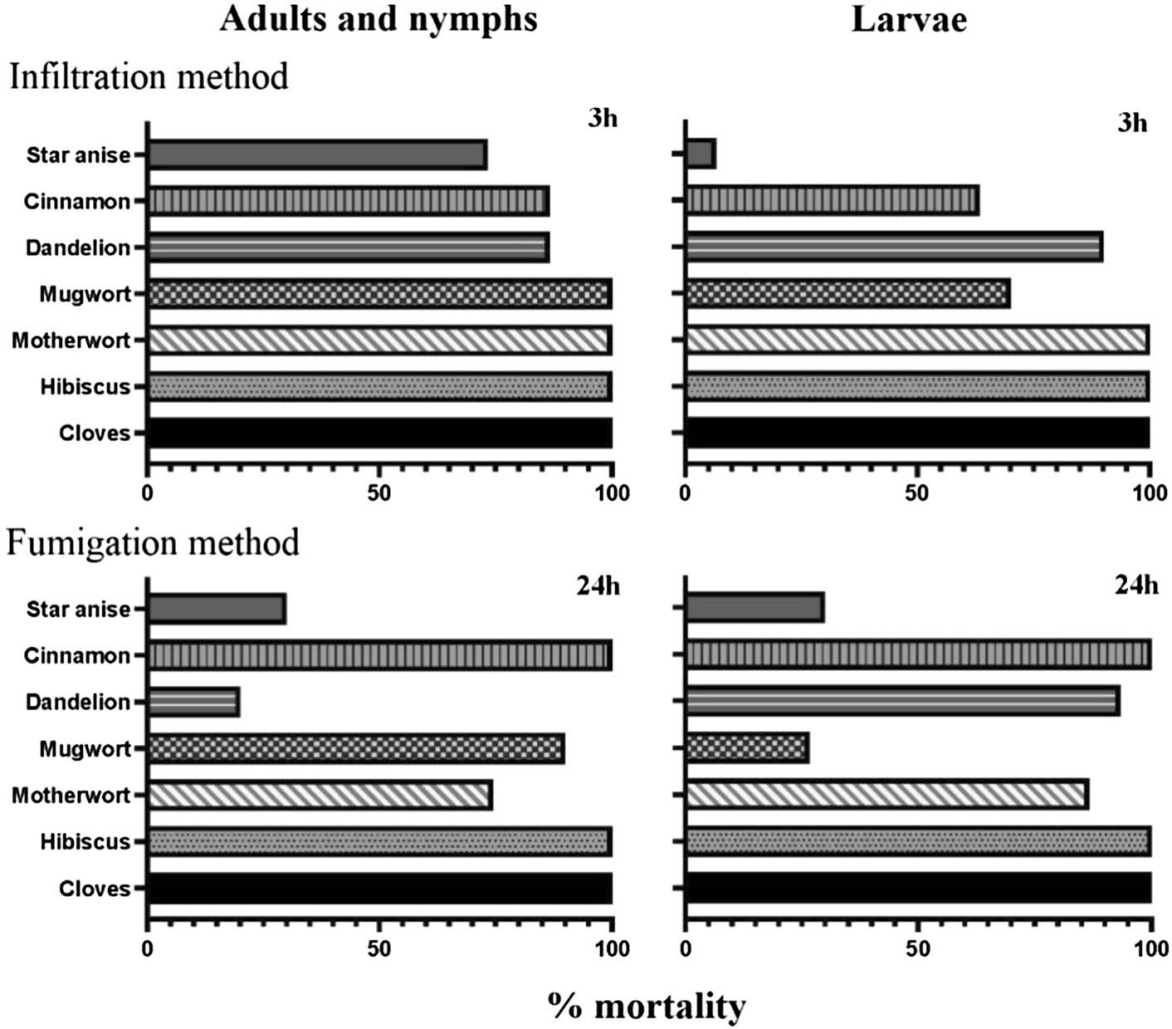
Comparison of in vitro acaricidal effects of ethanol extracts of seven herb extracts through fumigation and immersion on adults/nymphs and larvae of the northern fowl mite

In this study, the different administration methods and life stages significantly affected the mortality rate, as summarized in Fig. 2. The results showed that hibiscus, clove and motherwort had better acaricidal efficacy on NFMs using the infiltration method, whereas cinnamon had higher and more stable repellent activity on NFMs using the fumigation method. Among the herbs, star anise (infiltration) and Chinese mugwort (fumigation) only had acaricidal effects on the adult and nymphal stages of NFMs, and dandelion (fumigation) only on the larval stage. Clove and hibiscus had 100% mortality to adults, nymphs and larvae of NFMs using both infiltration and fumigation methods. This study is the first to find that motherwort and hibiscus have acaricidal activity against NFMs. In the in vitro evaluation of the effects of alcohol herb extracts on the egg hatching rate of the NFMs, after 48 h, Chinese mugwort was found to have the lowest hatching rate of 19%.

According to previous studies, terpenoids, flavonoids, sugars, phenols and alkaloids can inhibit the activity of acetylcholinesterase, causing toxicity in the mite nervous system. In addition, these chemicals can act on other targets in the mite nervous system such as nicotinic acetylcholine receptors (Nacr), octopamine receptors, tyramine receptors, sodium channels and gamma-aminobutyric acid (Gba)-gated chloride channels (Pritchard et al. 2015). These may be the mechanism of action of herbal remedies against mites, as the active ingredients in herbs are complex and abundant. These results have been confirmed in a number of studies on herbal mite control.

Lee et al. (2019) reported that clove contains a volatile oil (15–20%) and that this oil is mainly composed of eugenol (78%–95%), acetyl eugenol (7.3%), and ß-caryophyllene (9%). At a concentration of 1.3 µg/m^2^, it has a 100% acaricidal effect on *D. gallinae* (Lee et al. 2019; Tabari et al. 2020). In a contact test of the effects of plant essential oils on mites, clove was found to have the highest contact toxicity, with an LC_50_ value of 8.9 µg/mL; in addition, clove has been reported to have repellent and acaricidal activity on various arthropods, such as the whitefly *Bemisia tabaci* and the ticks *Rhipicephalus microplus* and *Dermacentor nitens* (Kim et al. 2011; Zeringota et al. 2013).

Studies have reported that Chinese mugwort contains a variety of biologically active compounds such as phenols, flavonoids, eucalyptol, β-caryophyllene, camphor and other components (Xiao-Yan et al. 2020). This herb has antibacterial and antiviral effects, can stop bleeding and pain, and can be used externally to reduce dampness and relieve itching (Dib and El Alaoui-Faris 2019). Chinese mugwort is widely used in China not only for moxibustion and foot bathing but also for consumption, as in the traditional Chinese food ‘Qing Tuan’. Chinese mugwort has often been reported to have insecticidal activity; for example, it has obvious acaricidal effect on *Demodex folliculorum*, *D. brevis* and the cabbage aphid *Brevicoryne brassicae* (Ahmed et al. 2020; Du et al. 2021). According to previous research (Zhang et al. 2014), the four compounds it contains (β-caryophyllene, eucalyptol, β-pinene and camphor) have strong acaricidal activity on adults of *Lasioderma serricorne*. These compounds might also act on NFM eggs, which would explain the inhibitory effect of Chinese mugwort found in the current study.

Hibiscus (Malvaceae) contains many components such as alkenes, esters, aldehydes, alcohol compounds, and alkanes (Wei et al. 2015). At present, there are no reports on the acaricidal effect of hibiscus. Only one study addresses its insecticidal effects. For the larvae and adults of the pollen-feeding phytoseiid mite *Amblyseius swirskii*, eating hibiscus pollen can cause 100% mortality (Goleva and Zebitz 2013).

Motherwort is mainly distributed in Russia and China. It promotes blood circulation, regulates menstruation, has detoxification effects, and reduces fever, diuresis, and swelling (Wojtyniak et al. 2013). A variety of compounds such as monoterpenes, flavonoids, phenolic acids, volatile oils, and sterols have been identified in motherwort (Shang et al. 2014). The acaricidal activity of motherwort has not been previously reported.

Many plant essential oils and herbal extracts contain various volatile compounds with fumigation effects such as alkanes, alcohols, aldehydes, and especially terpenes and monoterpenes (Bordin et al. 2021). The main component of cinnamon bark oil is cinnamaldehyde (Lee et al. 2019), which may explain the difference in NFMs mortality between the infiltration method (13% after 30 min exposure) and the fumigation method (87% after 6 h).

Therefore, when extracting herbs, a higher extracted content may result in a higher toxicity in mites. However, plant components may vary due to many factors, such as the plant location, extraction method, plant age, cultivation conditions and harvest time (Fernandes et al. 2019; JerkovicÂ and Milos 2001). For example, it has been reported that star anise exhibits a substantial mortality effect on *D. gallinae* (Tabari et al. 2020), but the effect observed in our experiment was not substantial. This may be related to the extraction method and place of origin.

## Conclusions

In this study, *S. aromaticum* (clove), *A. argyi* (Chinese mugwort), *H. syriacus* (hibiscus) and *L. artemisia* (motherwort) extracts caused significant mortality of *O. sylviarum* at different life stages. The mechanisms and compositions associated with the effects on *O. sylviarum* are currently unknown, so further in-depth study is needed. In addition, if these herbs are mixed at appropriate treatment dosages and used together with appropriate control methods (spraying or fumigation), greater mortality and stability could be achieved.
